# Lack of Causal Relationships Between Chronic Hepatitis C Virus Infection and Alzheimer’s Disease

**DOI:** 10.3389/fgene.2022.828827

**Published:** 2022-03-10

**Authors:** Lin Huang, Yongheng Wang, Yaqin Tang, Yijie He, Zhijie Han

**Affiliations:** ^1^ Department of Bioinformatics, School of Basic Medicine, Chongqing Medical University, Chongqing, China; ^2^ International Research Laboratory of Reproduction and Development, Chongqing Medical University, Chongqing, China

**Keywords:** chronic hepatitis C (HCV), Alzheimer disease, Mendelian randomization (MR) analysis, GWAS—genome-wide association study, cause

## Introduction

Inflammation produces hepatic encephalopathy in patients with chronic liver disease by modulating brain functions (O'Beirne et al., 2006). Several studies have reported that patients with chronic hepatitis C virus (HCV) infection tend to exhibit cognitive impairment and may increase the risk for dementia (Chiu et al., 2014; Adinolfi et al., 2015; Choi et al., 2021). Meanwhile, the HCV genome has been detected in the brain tissues of some patients with dementia, which suggests that it may be able to infect the central nervous system (CNS) directly (Forton et al., 2001; Khonsari et al., 2015). A recent study reported that treatment of HCV infection with direct-acting antivirals (e.g., glecaprevir/pibrentasvir, elbasvir/grazoprevir, and ledipasvir/sofosbuvir) significantly reduces mortality risk in patients with Alzheimer's disease (AD) and related dementia (Tran et al., 2021). Furthermore, apolipoprotein E (ApoE) plays a key role in the mechanism of AD by driving amyloid-β (Aβ) peptide accumulation in the brain (Yamazaki et al., 2019). Previous studies demonstrated that the ApoE level affects HCV infection and action in the CNS by regulating the blood–brain barrier permeability and is significantly associated with the neuropsychiatric symptoms in HCV-infected individuals (Gochee et al., 2004; Sheridan et al., 2014; Wozniak et al., 2016). Although evidence has shown that HCV infection is associated with the dysfunctions of the CNS, it is not clear whether any HCV infection influences AD pathogenesis. Observational studies are difficult to interpret because these results may have been influenced by reverse causation and confounding factors. Mendelian randomization (MR) has the potential to evaluate causal relationships between exposure and outcome in the presence of such limitations (Sekula et al., 2016; Davies et al., 2018). In this study, we investigated the causal impact of HCV infection on the risk of late-onset AD by implementing Causal Analysis Using Summary Effect estimates (CAUSE), a novel MR method that can avoid more false positives caused by correlated horizontal pleiotropy (Morrison et al., 2020).

## Analysis of Association Between HCV Infection and Risk of Late-Onset AD

The summary data for exposure (HCV infection) was downloaded from the National Bioscience Database Center (NBDC) Human Database which includes the complete results of genome-wide association studies (GWAS) based on 5,794 HCV susceptible cases and 206,659 controls (NBDC Research ID: hum0014.v17.CHC.v1) (Ishigaki et al., 2020). The GWAS results of late-onset AD were obtained from the International Genomics of Alzheimer's Project (IGAP) (*n* = 17,008 late-onset AD cases and 37,154 controls) (Lambert et al., 2013). In addition, Manhattan plot of HCV and AD GWAS results are in Supplementary Figure S1. According to the manual of CAUSE, there should be as many single-nucleotide polymorphisms (SNPs) as the instrumental variable (IV) to estimate CAUSE posteriors to ensure the accuracy of the MR results (https://github.com/jean997/cause). Therefore, we used the more liberal threshold of the GWAS significance and the independence of SNPs (i.e., *p* < 0.001 and *r* ^2^ < 0.01, respectively) in this study. The “'ld_clump” function of the R package “ieugwasr” was used to calculate pairwise prune linkage disequilibrium (LD) measures between these SNPs based on the 1000 Genomes project phase I and prune the non-independent ones (https://mrcieu.github.io/ieugwasr/). To avoid the pleiotropy effects, CAUSE included as many information from all variants as possible, even weakly associated variants. It computed the test statistic to distinguish variants associated with confounders. At last, we use two-sample MR for further validation (Morrison et al., 2020).

After merging the GWAS results between the NBDC HCV and IGAP AD studies, and further removing the variants with ambiguous and mismatched alleles, we selected 4,289,211 SNPs which match both datasets for the following analyses. The nuisance parameters are estimated by finding the mixing parameters and the maximum a posteriori estimate. According to the significance threshold of the GWAS *p* < 0.001 and the LD analysis *r* ^2^ < 0.01, there are a total of 606 SNPs as IVs for the CAUSE fitting. As Table 1 shows, when compared with the null model, the estimated difference in expected log pointwise posterior density (delta EPLD) in both the sharing and causal models is positive, and no significant differences are presented (*p*-value is 0.95 and 0.83, respectively). Further, the fitted delta EPLD of the causal model is still not significantly better than the sharing model (z-score = 0.80 and *p*-value = 0.79). This revealed a similar posterior distributions and proportion of correlated pleiotropic SNPs in the two models. A total of 42 SNPs for any HCV infection were identified (Supplementary Table S1). Two-sample MR analysis indicated that genetically predicted HCV infection was not associated with AD (odds ratio (OR) = 0.99, 95% confidence interval (CI) = −0.07to 0.04, *p* = 0.69) (Supplementary Table S2). The resulting estimates of the effect of HCV on AD are shown in the scatter plot (Supplementary Figure S2). Generalized funnel plot indicated the absence of directional pleiotropy (Supplementary Figure S3). Leave-one-out analysis revealed a high stability of our results (Supplementary Figure S4). Thus, the present MR study affords no support for causality between HCV and AD, which suggests that HCV infection has no influence on the late-onset AD.

**TABLE 1:** The results of causality of HCV infection and AD by comparing causal and sharing model.

Model 1	Model 2	Δ ELPD	s.e. Δ ELPD	Z-Score	*p*-value
Null	Sharing	0.22	0.13	1.70	0.95
Null	Causal	0.96	1.00	0.94	0.83
Sharing	Causal	0.74	0.92	0.80	0.79

Model 1 and Model 2 imply the models being compared. When estimated difference in ELPD (Δ ELPD) = ELPD_C_ − ELPD_S_ is negative, model 2 is a better fit.

Key: s.e. Δ ELPD, estimated standard error of Δ ELPD; Z-Score, Δ ELPD/s.e. Δ ELPD.

## Discussion

The traditional MR methods may tend to lead to false positive results because the assumption about horizontal pleiotropy of instrument is often violated. Therefore, in this study, using the largest available GWAS results on HCV infection and AD, we investigated a potential causal role for HCV infection in late-onset AD using an improved MR analysis. However, the results failed to reveal any causal association between them, which appears to be in conflict with some previous reports about the influence of HCV infection on the human CNS (Weissenborn et al., 2004; Yarlott et al., 2017). Previous studies demonstrate that ApoE and its cell-surface receptor is a key prerequisite for HCV production and infectivity by enriching the virus particles and giving rise to the lipoviral particles hybrid with lipoproteins. Given that ApoE also plays an important role in Aβ peptide accumulation, a potential explanation for these findings could be that the ApoE level may affect HCV infection and also mediate the genetic risk of late-onset AD in patients with HCV and AD (Jiang and Luo, 2009; Hishiki et al., 2010; Yang et al., 2016) and thus leads to a false association between HCV infection and AD. In conclusion, our MR analyses found no evidence for a causal role of HCV for late-onset AD pathogenesis. These findings could further improve the conclusions of previous studies, and further research are needed to elucidate the underlying mechanisms.

## Conclusion

In this study, we used both CAUSE and two-sample MR study to assess the causal effects of HCV infection on AD. The CAUSE results revealed that HCV infection did not appear to have a causal effect on the risk of AD. Similar trends were observed through two-sample MR. The evidence suggests that previously reported observational associations could have resulted from confounding. Future studies are warranted to clarify the underlying mechanism.
