# Supplementary material for: Lack of Causal Relationships Between Chronic Hepatitis C Virus Infection and Alzheimer’s Disease
Source: Front Genet. 2022 Mar 10;13:828827. doi: 10.3389/fgene.2022.828827 (PMC8959984; doi:10.3389/fgene.2022.828827)
Supplement: Supplementary file 1 [file DataSheet1.PDF]

Supplementary Table 1 Single nucleotide polymorphisms (SNPs) considerably (P-value<1e-3) and independently (r2<0.01) associated with Chronic Hepatitis C virus (HCV) infection (42 SNPs)

SNP	Chr	Position	EA/OA	Beta	SE	*P*-value
rs10748931	10	108811603	A/C	-0.11306	0.033295	6.85E-04
rs10787286	10	112623274	C/A	0.179479	0.04746	1.56E-04
rs10828832	10	25863964	A/C	0.091304	0.026061	4.59E-04
rs11222181	11	130441491	C/T	-0.10849	0.030745	4.18E-04
rs11244857	10	127851943	G/A	0.077436	0.021836	3.91E-04
rs117146098	10	79820935	C/T	0.083529	0.023835	4.58E-04
rs12264935	10	106522667	C/T	-0.07159	0.018912	1.54E-04
rs12266595	10	17507136	T/C	-0.06898	0.019134	3.12E-04
rs12277819	11	122586944	A/G	0.143768	0.043568	9.67E-04
rs12577018	11	21064322	C/T	-0.09651	0.028914	8.45E-04
rs1274376	10	92233681	A/C	-0.06812	0.020507	8.94E-04
rs142233599	11	26351389	T/C	-0.11097	0.033108	8.03E-04
rs142416119	10	104966698	C/T	-0.12532	0.027052	3.61E-06
rs17310069	11	28133420	A/G	-0.09057	0.026727	7.03E-04
rs1903857	10	127881055	T/C	-0.06552	0.019438	7.50E-04
rs196312	10	121382723	G/A	-0.074	0.021479	5.70E-04
rs2038558	10	37877402	G/A	-0.07314	0.020398	3.36E-04
rs2038657	10	77665764	T/C	0.078712	0.020298	1.05E-04
rs2449095	11	107524585	C/T	0.071104	0.019093	1.96E-04
rs2729854	11	19412342	A/G	0.069934	0.019647	3.71E-04
rs3781180	10	79742617	G/T	0.081656	0.024711	9.52E-04
rs4747209	10	73611916	C/T	0.081347	0.023228	4.62E-04
rs4749791	10	8633861	C/T	0.068386	0.019652	5.02E-04
rs4751876	10	123897166	T/C	0.079351	0.023407	6.99E-04

Supplementary Table 1 (cont'd)

SNP	Chr	Position	EA/OA	Beta	SE	*P*-value
rs608006	11	126469558	T/C	0.083591	0.018958	1.04E-05
rs61854767	10	5455019	G/T	0.09743	0.025383	1.24E-04
rs621106	10	103065599	G/A	-0.07597	0.021558	4.25E-04
rs6537581	10	50046120	T/C	0.081053	0.02099	1.13E-04
rs66495925	11	122575256	C/T	0.145657	0.042509	6.11E-04
rs683387	11	103311819	T/G	-0.07113	0.021444	9.09E-04
rs7090827	10	12111789	C/T	0.145694	0.038413	1.49E-04
rs7092524	10	18987158	A/C	0.065237	0.019195	6.77E-04
rs7102941	11	114023607	T/C	0.24516	0.072195	6.84E-04
rs72638745	10	8685074	G/A	0.114961	0.031313	2.41E-04
rs74147831	10	73068779	A/G	-0.11192	0.030544	2.48E-04
rs7894390	10	12543415	G/A	-0.06338	0.019024	8.64E-04
rs78972616	10	8493087	C/T	-0.12965	0.034978	2.10E-04
rs7909761	10	113124015	A/G	-0.07235	0.02133	6.94E-04
rs827913	10	9389421	T/G	-0.06802	0.019616	5.26E-04
rs893500	10	133005521	G/A	0.590216	0.169748	5.07E-04
rs903020	11	130312803	G/A	-0.10016	0.029944	8.23E-04
rs949295	11	120789852	T/C	-0.06665	0.019077	4.77E-04

Abbreviations: SNP, single nucleotide polymorphism; Chr, chromosome; EA effect allele; OA other allele; SE standard error

Supplementary Table 2 Association of genetically predicted Chronic Hepatitis C virus (HCV) infection (*P*-value<1e-3 and r^2<0.01) with Alzheimer's disease (AD) using Mendelian randomization (MR) with different methods.

SNPs	Method	beta	OR	95% CI		*P*-value	IVW		MR-Egger	
							Cochran's *Q*-statistic	P-value	Intercept	*P*-value
42	MR Egger	0.023	1.023	-0.068	0.113	0.627	49.640	0.167	-0.005	0.368
	Weighted median	0.022	1.022	-0.068	0.112	0.634				
	Inverse variance weighted	-0.011	0.989	-0.066	0.044	0.693				
	Simple mode	-0.042	0.958	-0.201	0.117	0.604				
	Weighted mode	0.018	1.018	-0.054	0.090	0.633				

Abbreviations: OR, Odds Ratio; CI, confidence interval.

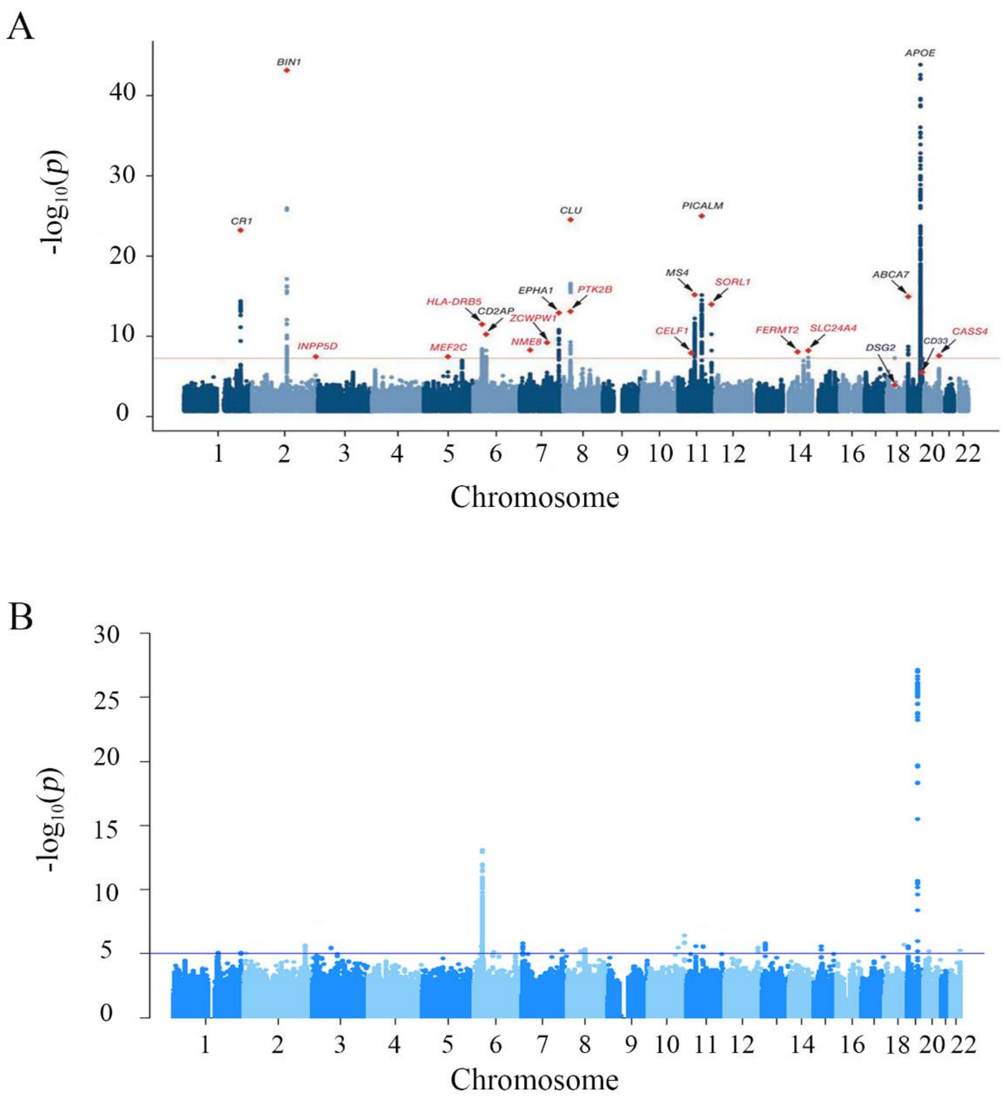

Supplementary Figure 1 The Manhattan plots of a genome-wide association study (GWAS) for HCV infection (A) and AD (B). (A) Manhattan plot of stage 1 for genome-wide association with Alzheimer's disease (17,008 cases and 37,154 controls).

Genome-wide thresholds for significant (P = 5E-08) is indicated by the grey line. Genes previously identified by GWAS are shown in black, and newly associated genes are shown in red. (B) Manhattan plot of genome-wide association study (GWAS) with HCV infection (5,794 cases and 206,659 controls). Genome-wide thresholds for significant (P = 5E-05) is indicated by the blue line.

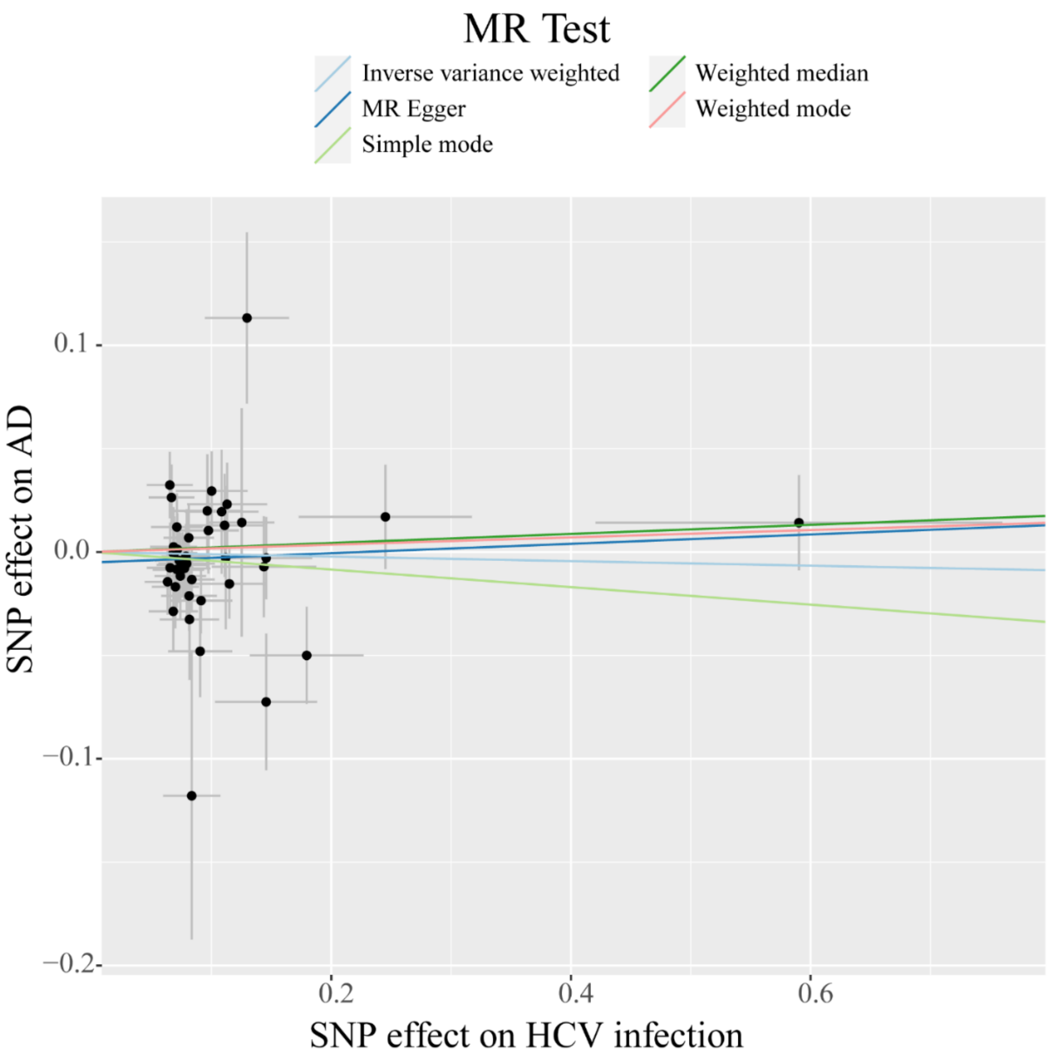

Supplementary Figure 2 Scatter plots of the 5MR models for HCV infection with potential causal relationship with AD. vertical and horizontal grey lines show the 95% confidence intervals (CI) for each SNP. The slope of the red solid line corresponds to the Mendelian randomization (MR) estimate.

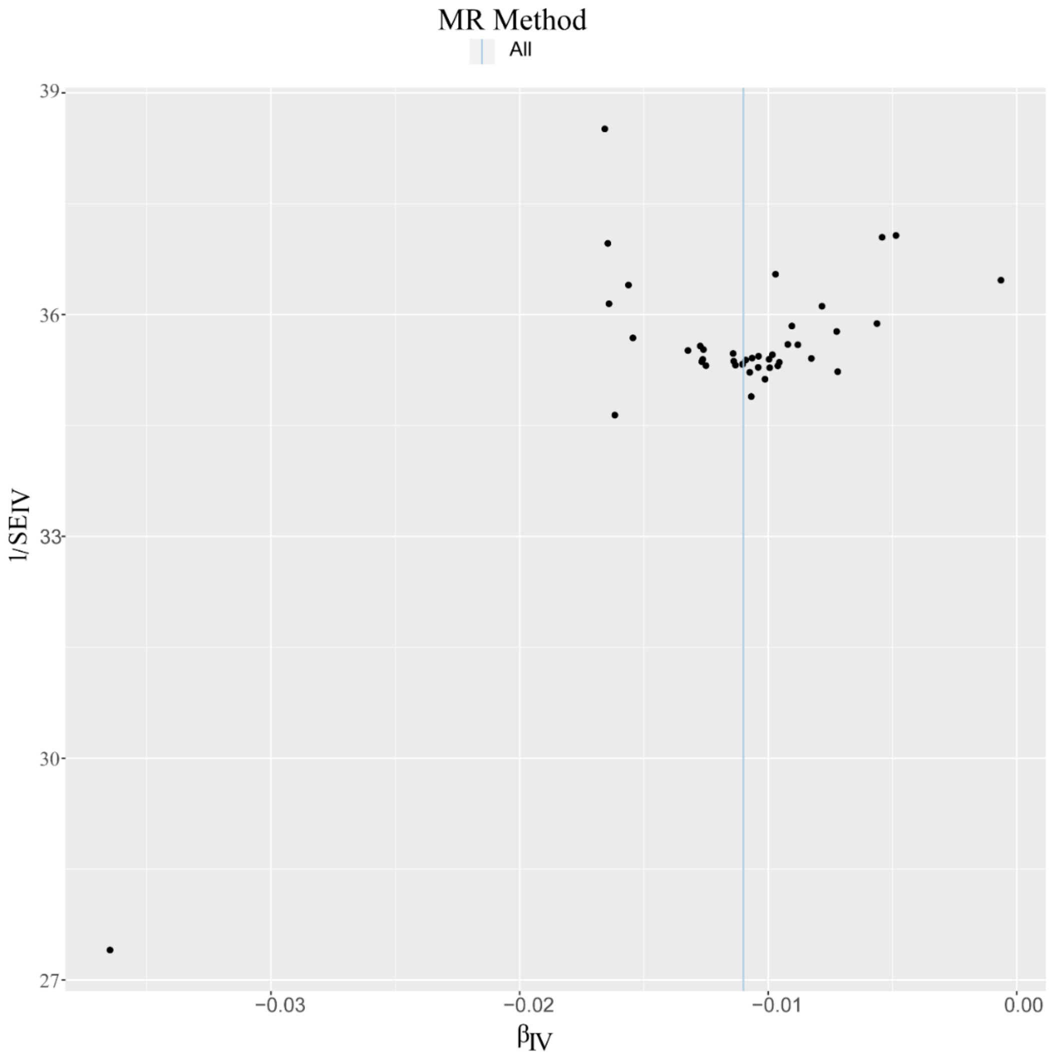

Supplementary Figure 3 Generalized funnel plot of MR analysis from HCV infection on AD. Funnel plots offer a simple way to detect directional pleiotropy. As can be appreciated from the plot, no evidence for asymmetry was found, with no indication of pleiotropy.

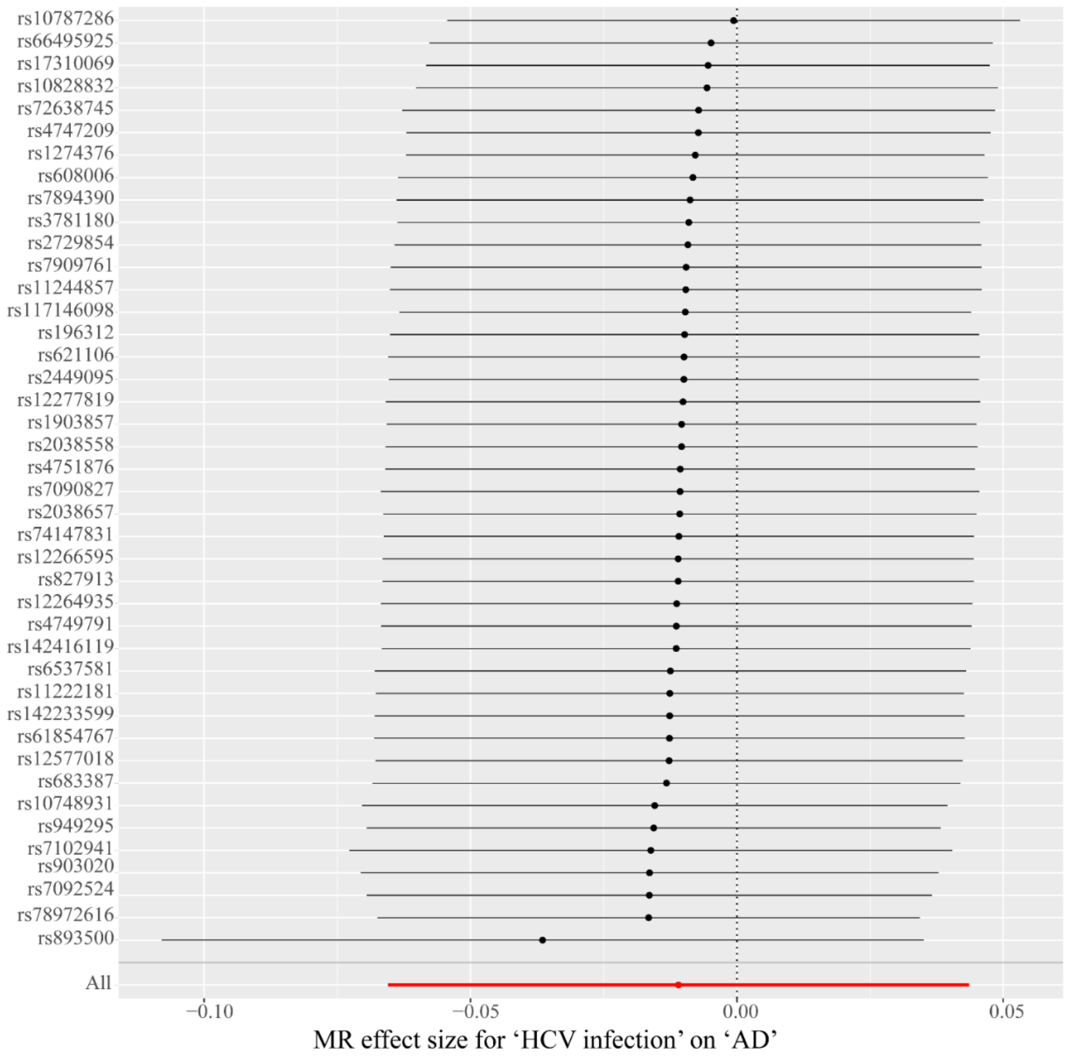

Supplementary Figure 4 Leave-one-out sensitivity of MR analysis from HCV infection on AD. Leave-one-out analysis is used to evaluate whether any single instrumental variable was driving the causal association of HCV infection with AD.
